# Supplementary material for: The role of International Civil Society Organizations in democratization: A crisp-set QCA approach to anti-corruption in Ghana
Source: PLoS One. 2023 Nov 17;18(11):e0291388. doi: 10.1371/journal.pone.0291388 (PMC10655974; doi:10.1371/journal.pone.0291388)
Supplement: S1 File — (DOCX) [file pone.0291388.s001.docx]

Dear Respondent; Civil Society Organization,

We appreciate your attention and consideration to participate in our study. My name is Ebenezer Kurtis Graham in affiliation with the Department of International Relations, University of Cape Coast Ghana, and my co-author is Dr. Emmanuel Oluwatosin Adewusi, Bahçeşehir Cyprus University, Political Science and International Relations Department.

We are conducting a survey on the “The role of International Civil Society Organizations in democratization: A crisp-set QCA approach to Anti-corruption in Ghana” and would be appreciate a response from your administrator.

We kindly request your consensual participation in responding to the questions below. We declare that the information given will be treated as confidential and the responses will be used in this research only and only for academic research purposes only.

Thank you for your time.

| **Part A: Demographic Information:**   1. **Age of your organizations…………** 2. **Region of organization……………** 3. **Areas of focus: …………….** 4. **Affiliation to ICSO: Oxfam 🞏 Transparency International  Amnesty International  Global Organization of Parliamentarians against Corruption (GOPAC)  Others…..** 5. **Position in the CSO …………………………………………………………………** | | | | | |
| --- | --- | --- | --- | --- | --- |
| **Item** | **SD** | **D** | **N** | **A** | **SA** |
| **Role of international civil society organization** | | | | | |
| **Strength of international civil society have a positive influence on the state and the market** |  |  |  |  |  |
| **The international civil society mobilize particular constituencies, vulnerable and marginalized sections of masses, to participate more fully in politics and public affairs** |  |  |  |  |  |
| **Policy analysis and advocacy can be assisted by the international civil society** |  |  |  |  |  |
| **The international civil society is an important agent for promoting good governance like transparency, effectiveness, openness, responsiveness and accountability** |  |  |  |  |  |
| **The international civil society building social capital and enabling citizens to identify and articulate their values, beliefs, civic norms and democratic practices** |  |  |  |  |  |
| **Anti-corruption** | | | | | |
| **People should act positively and cooperate** |  |  |  |  |  |
| **People should be praised for working honestly** |  |  |  |  |  |
| **Proving corruption free environment is important** |  |  |  |  |  |
| **Freedom of expression** | | | | | |
| **There is a free and independent media and other forms of free cultural expression** |  |  |  |  |  |
| **There are free religious institutions, and there are free private and public religious expression** |  |  |  |  |  |
| **There is open and free private discussion** |  |  |  |  |  |
| **Social trust** | | | | | |
| **People expect to get rewards when trusting others** |  |  |  |  |  |
| **People in our country are credible** |  |  |  |  |  |
| **The rule of law works in our country** |  |  |  |  |  |
| **It is beneficial to believe in people to start something in Ghana** |  |  |  |  |  |
| **Leadership** | | | | | |
| **Remind each other to work fairly and honestly** |  |  |  |  |  |
| **Praise for working honestly** |  |  |  |  |  |
| **Act decisively when anti-corruption issue is raised** |  |  |  |  |  |
| **Rules and Regulations** | | | | | |
| **Rules protect us from vigilance cases** |  |  |  |  |  |
| **Rules should be consulted by all** |  |  |  |  |  |
| **Fears of Punishment** | | | | | |
| **Fear of disciplinary action** |  |  |  |  |  |
| **Fear of suspension** |  |  |  |  |  |
| **Training** | | | | | |
| **Training is necessary** |  |  |  |  |  |
| **Training helps in prevention of corrupt practices** |  |  |  |  |  |
