# Supplementary material for: The role of International Civil Society Organizations in democratization: A crisp-set QCA approach to anti-corruption in Ghana
Source: PLoS One. 2023 Nov 17;18(11):e0291388. doi: 10.1371/journal.pone.0291388 (PMC10655974; doi:10.1371/journal.pone.0291388)
Supplement: Attachment — Submitted filename: Response to Reviewers.docx [file pone.0291388.s002.docx]

Dear Editor & Reviewers,

Thank you for reviewing our manuscript and allowing us to revise our work to meet the journal's standards and address the reviewers' comments.

We would also like to thank you for extending the submission period.

We have attended to the comments of the Editor and the reviewers. Kindly find below our responses which addressed the comments and have been appropriately integrated into the main study at a glance per the attached.

We look forward to your feedback on our study.

Thank you.

Best Regards,

Signed

Ebenezer Kurtis Graham

Ozker Kocadal

Att:

**Responses to the Editor’s comment**

Thank you for your comments to enhance our article to meet the journal standards.

The PLOS ONE format link has been used to prepare this current manuscript following the journal's style.

The minimum data for this analysis has been made available using the Kaggle for replication, it has been cited in the manuscript, and the doi has been inserted below the tables. All tables have also been referred to in the presentation of the results.

The ORCID for the corresponding author has been linked to the submission profile according to the request of the journal.

There are no supporting files, as all files have been made available on Kaggle. 10.34740/kaggle/dsv/5789902

**Response to Reviewer 1**

Thank you for your comments for improving on our research.

The objective has been rewritten in the new introduction with more details of the research questions. The scope of the bibliography has been broadened as sources for the manuscript have been increased to 53 as recommended by the reviewer. In the discussion, a comparative discussion was made concerning Nigeria and Kenya’s anti-corruption strategy. The discussion stands alone, while the conclusion and recommendations pointing to the applicability of the findings are the last sections.

**Response to Reviewer 2**

Thank you for your comments on improving our study.

Firstly, the structural problems identified in the previous version have been addressed. The manuscript introduction has been improved, addressing corruption and examining possible anti-corruption strategies. The previous introduction roamed democracy; as you noted unnecessary to roam around that. However, the current version quickly touches upon democracy only in connection to corruption. The structure of the paper has also been included in the last paragraph of the article. However, due to the broadening of the literature review, as suggested by Reviewer 1, the length of the introduction has only been shortened reasonably.

It identified the problems in the case of Ghana, examined the literature that suggested different conditions, and highlighted the limitation of regression analysis. It concludes by stating the objective, the research questions, and the hypothesis. This resolves the length of the introduction and the repetitions in the introduction.

The connection of CSO and ICSO has been highlighted in the introduction.“Some studies suggest that International Civil Society Organizations (ICSOs) significantly strengthen local CSOs to overcome challenges by effectively mobilizing skills and financial resources. The CSO receives technical support from ICSO when facing challenges that limit its operation. The ICSO supports the CSO with funding, advocacy on issues being suppressed by the government, technical advice, and networking; it connects the CSO to a network that can provide other needed support for effectiveness. This study examined the CSO evaluation of the ICSO such as Transparency International, Amnesty International, and the Global Organization of Parliamentarians against Corruption (GOPAC), which supports CSO and pushes the government to address corruption”.

The seven conditions have been chosen based on the large number of respondents thus, the more the cases the more the respondents. “Seven conditions were chosen in compliment to the size of the population examined. According to Ragin, Schneider, and Wagemann, when the population is large, more conditions are needed to provide the diversity of explanatory factors that would bring about more configuration. Since the population size is 264, more factors are needed for the analysis”.

Your suggestion to change cases to respondents has been applied to the study as suggested since the study's main objective is to identify configurations. However, regression analysis does not provide the interaction among conditions to bring out an outcome, i.e., configuration. Hence, csQCA has been maintained.

The data was collected based on demography because domestic CSO in Ghana has faced different challenges based on location. For example, the Northern CSO faced insecurity due to the corrupt practice of politicians who have failed to pursue infrastructure that deter bandits and nomads from attacking the people. While in the South, insecurity is based on natural disasters due to embezzlement of public funds. Other CSOs also experience the impact of corruption differently. Therefore, demography is a justified pattern of data collection rather than the capacity of the CSO because it represents the strategies CSOs suggested for anti-corruption by all-district.

Pappas and Woodside stated the feasibility of adopting the Likert scale into QCA. The Likert scale was adapted to csQCA using the STATA version 13 to generate variables to calculate the average of all questions that measure each variable. Following Parker’s study on analyzing criminology through csQCA, which used the arithmetic mean as the cut-off point when applying the Likert scale, our analysis also used the mean for the cut-off point to set membership score. Membership in conditions was calibrated as 0 for non-membership in condition/variable and 1 for the membership in the condition/variable according to the following cut-off; anti-corruption strategy 3.67, training 4.0, fear of punishment 3.67, rule and regulations 4.0, leadership 3.67, social trust 3.75, Freedom of Expression 3.67 and RICSO 4.0. Grimes’ study on the success of civil society in combating corruption and Dixit’s study on anti-corruption institutions provide the expectation that these conditions and outcomes have an interaction that defines an effective strategy. Therefore, an anti-corruption strategy is considered adequate if the outcome receives 1 and ineffective if it receives 0 based on the interaction of the factors. The necessary condition analysis, the truth table, and the configurations were conducted using QCA version 3.1b software.

The presentation of the methodology has been rewritten to highlight how we arrived at our conclusion. The missing point was how the Likert scale was converted and used for csQCA, which has been explained in the methodology section following best practices in the literature. STATA generate variable command was used to get the average score of each respondent to the questions of the variables, which was calibrated based on cut-off point into binary coding.

Consistency and Coverage have been explained, i.e., consistency is the level of similar composition brought about the same result while Coverage is the percentage of the cases, in this case, respondents, covered by the solution.

Regarding social trust, in the first configuration of an effective anti-corruption strategy, social trust was present (It was written as CS instead of ST, leading to misinterpretation); hence social trust is a significant factor present in 40 per cent of the cases analyzed. This has been corrected in the explanation of Table 3 and reflected in the explanation.

Discussion and Conclusion in the previous manuscript have been split into two sections; 1) Discussion and 2) Conclusion and Recommendation. These sections have been written carefully to engage readers that might not be conversant with csQCA.

The following paragraph below has been added to address the “So what” question.

While CSO continues to look outward for support from ICSO, this study recommends that they continue to seek to strengthen the domestic mechanisms; freedom of expression, social trust, leadership, social trust, rules, regulation, and training for effective anti-corruption strategy, and getting government officials and politicians to say yes to becoming more committed to being accountable rather than seeing them as a tool used by developed countries. This conclusion is arrived at because ICSO cannot function without some domestic mechanisms being put in place. In this case, therefore, leadership against corruption and the fear of punishment for all corrupt conduct is activated.

Typographical errors have been addressed, as well as inconsistent acronyms, especially in the case of RICSO. Lastly, additional references have been added.
